# AI-Augmented Point of Care Ultrasound in Intensive Care Unit Patients: Can Novices Perform a “Basic Echo” to Estimate Left Ventricular Ejection Fraction in This Acute-Care Setting?

**DOI:** 10.3390/jcm14092899

**Published:** 2025-04-23

**Authors:** Cassandra Gallant, Lori Bernard, Cherise Kwok, Stephanie Wichuk, Michelle Noga, Kumaradevan Punithakumar, Abhilash Hareendranathan, Harald Becher, Brian Buchanan, Jacob L. Jaremko

**Affiliations:** 1 Department of Radiology and Diagnostic Imaging, Faculty of Medicine and Dentistry, University of Alberta, Edmonton, AB T6G 2R3, Canada; lbernar1@ualberta.ca (L.B.); ctkwok@ualberta.ca (C.K.); sawichuk@ualberta.ca (S.W.); mnoga@ualberta.ca (M.N.); punithak@ualberta.ca (K.P.); hareendr@ualberta.ca (A.H.); 2 Department of Medicine, Faculty of Medicine and Dentistry, University of Alberta, Edmonton, AB T6G 2R3, Canada; harald@ualberta.ca; 3 Department of Critical Care Medicine, Faculty of Medicine and Dentistry University of Alberta, Edmonton, AB T6G 2R3, Canada; bmb@ualberta.ca

**Keywords:** point-of-care ultrasound, artificial intelligence, left ventricular ejection fraction, echocardiography, critical care

## Abstract

**Background:** Echocardiography is crucial to understanding cardiac function in the Intensive Care Unit (ICU), often by measuring the left ventricular ejection fraction (LVEF). Traditionally, measures of LVEF are completed as part of a comprehensive examination by an expert sonographer or cardiologist, but front-line practitioners increasingly perform focused point-of-care estimates of LVEF while managing life-threatening illness. The two main echocardiographic windows used to grossly estimate LVEF are parasternal and apical windows. Artificial intelligence (AI) algorithms have recently been developed to assist non-experts in obtaining and interpreting point-of-care ultrasound (POCUS) echo images. We tested the feasibility, accuracy and reliability of novice users estimating LVEF using POCUS-AI echo. **Methods:** A total of 30 novice users (most never holding an ultrasound probe before) received 2 h of instruction, then scanned ICU patients (10 patients, 80 scans) using the Exo Iris POCUS probe with AI guidance tool. They were permitted up to 5 min to attempt parasternal long axis (PLAX) and apical 4 chamber (A4C) views. AI-reported LVEF results from these scans were compared to gold-standard LVEF obtained by an expert echo sonographer. To further assess accuracy, this sonographer also scanned another 65 patients using Exo Iris POCUS-AI vs. conventional protocol. **Results:** Novices obtained images sufficient to estimate LVEF in 96% of patients in <5 min. Novices obtained PLAX views significantly faster than A4C (1.5 min vs. 2.3 min). Inter-rater reliability of LVEF estimation was very high (ICC 0.88–0.94) whether images were obtained by novices or experts. In n = 65 patients, POCUS-AI LVEF was highly specific for a decreased LVEF ≤ 40% (SP = 90% for PLAX) but only moderately sensitive (SN = 56–70%). **Conclusions**: Estimating cardiac LVEF from AI-enhanced POCUS is highly feasible even for novices in ICU settings, particularly using the PLAX view. POCUS-AI LVEF results were highly consistent whether performed by novice or expert. When AI detected a decreased LVEF, it was highly accurate, although a normal LVEF reported by POCUS-AI was not necessarily reassuring. This POCUS-AI tool could be clinically useful to rapidly confirm a suspected low LVEF in an ICU patient. Further improvements to sensitivity for low LVEF are needed.

## 1. Introduction

Recent advancements in portable ultrasound technology are transforming the care of critically ill patients, including echocardiography. Echocardiography is a vital diagnostic tool in cardiovascular medicine, providing non-invasive insights into the heart’s structure and function [1]. Yet, as the demand for high-quality “echo” examinations continues to blossom, the human and technological resources needed to deliver such exams are finite [2]. Point-of-care ultrasound (POCUS) is an approach whereby treating clinicians apply focused echocardiography to quickly evaluate gross cardiac function, identify pericardial effusions, and assess response to fluids [3]. However, the effectiveness of POCUS in such settings hinges on the operator’s ability to obtain diagnostic-quality images and accurately interpret the findings [4]. In settings such as the emergency department or intensive care, practitioners of varying experience levels encounter innumerable obstacles to acquiring high-quality images [5,6,7].

The left ventricular ejection fraction (LVEF) is a key metric for cardiovascular assessment on echocardiography [8], measuring the percentage of blood ejected from the left ventricle during systole. LVEF offers critical insights into overall cardiac function and is essential for diagnosing and managing various cardiac conditions. The two principal echocardiographic windows used to measure LVEF are parasternal (i.e., parasternal long axis view; PLAX; parasternal short axis view; PSAX) and the apical windows (i.e., apical 4-chamber view; A4C; apical 2-chamber view; A2C). Obtaining these views traditionally requires a high level of technical expertise and dependable equipment to ensure diagnostic-quality images [8]. In emergency and critical care, factors such as patient instability, wounds, dressings, and invasive devices combine with body habitus and limited positioning challenge even the most skilled sonographer [9,10,11].

In helping to circumvent operator variability and patient limitations, one specific technology has found itself well-positioned to accelerate advancements in ultrasound imaging–artificial intelligence (AI). By automating key aspects of image acquisition and interpretation, AI has the potential to enable users at any skill level to perform complex cardiac assessments, including LVEF assessment, with minimal training [4,12]. Previous studies have also demonstrated that AI-based echocardiography tools can effectively calculate LVEF from both parasternal and apical views [13,14]. However, no research to date has evaluated the application of POCUS-AI tools in acute care settings. This capability is particularly important in high-stakes environments like the intensive care unit (ICU), where rapid and accurate decision-making is needed to positively impact patient outcomes. If performed well, the integration of AI-driven tools such as LVEF calculation could reduce the barriers to the performance and interpretation of echocardiography and better inform acute care.

This study seeks to evaluate the feasibility, reliability and accuracy of POCUS-AI systems in an intensive-care setting by addressing two key hypotheses: (1) novice users can reliably acquire both diagnostic-quality PLAX and A4C views in ICU patients, and (2) the accuracy of AI-calculated LVEF derived from both of these views is not inferior to conventional echocardiography performed concurrently by expert sonographer.

## 2. Materials and Methods

### 2.1. Study Design

We performed a prospective observational study with institutional ethics approval in place (University of Alberta HREB Pro00119711). Ethical protocols ensured that the novice users of the ultrasound-AI tool provided informed consent to participate, while scans of ICU patients (who were generally unconscious and intubated) were integrated into routine care on a waiver-of-consent basis, as echo is routinely performed in the ICU setting with minimal potential harm.

### 2.2. Hardware and Software

We scanned patients using the Exo Iris portable ultrasound probe with ExoAI software, a commercially available tool developed by Exo Inc. (Santa Clara, CA, USA, version 2.1.0). ExoAI has two different LVEF analysis packages for PLAX and A4C views. Although we received periodic updates to the software user interface, the AI algorithm was not actively retrained or fine-tuned on the data we collected during the course of the study.

### 2.3. Study Participants

Participants performing ultrasound scans included an expert professional echo sonographer with 26 years of experience, who also collected our gold-standard images and multiple novice learners. Inclusion criteria for learners included healthcare professionals (typically nurses, medical students, or physicians in training), graduate students/research assistants with a health-sciences project focus, the ability to provide written informed consent to be trained to perform basic echocardiography, and sufficient time to perform multiple scans. We excluded learners who had prior formal imaging experience (e.g., sonographers and radiologists). We recruited 30 novice learners (7 medical students, 2 graduate students, 21 resident physicians).

### 2.4. Patients

We included 75 consecutive ICU patients who were receiving conventional echocardiography as part of their care. These were divided into a small cohort (Cohort 1, n = 10), who were consecutively selected without regard for cardiac function, for whom novice scanners were available to provide measurements in addition to an expert sonographer, and a larger cohort (Cohort 2, n = 65), where only the expert sonographer provided measurements (Table 1). We included adult patients with a wide range of cardiac and intrathoracic pathologies of any age [mean (SD) Cohort 1: 57.6 (19.7); Cohort 2: 64.3 (10.8)], sex (Cohort 1: 70% M; Cohort 2: 71% M), ethnicity, height, weight, and body mass index (BMI) [mean (SD) Cohort 1: 27.9 (4.8); Cohort 2: 27.2 (5.0)] We excluded patients too unstable to safely delay care for a 15-min research ultrasound, congenital structural cardiac anomalies, and those currently in isolation for COVID-19 or other communicable diseases. Allowing for this, the patients in Cohort 1 and Cohort 2 were felt to represent a typical cross-section of ICU patients seen in our institution.

### 2.5. Training

This was intentionally kept brief. Novice learners underwent a standardized two-hour training session that included a comprehensive presentation on ultrasound cardiac imaging and techniques for obtaining parasternal and apical views. This session covered topics on basic ultrasound principles, cardiac anatomy, and imaging landmarks. Additionally, the training included one hour of hands-on practice, during which learners received direct guidance from an expert sonographer to refine their skills and confidence.

### 2.6. Scan Protocol

Most echocardiographic examinations in this study were conducted with the patient in the supine, semi-supine, or partial left decubitus position, depending on patient-related factors (i.e., clinical stability, spinal stabilization, external fixators).

**Figure 1:**
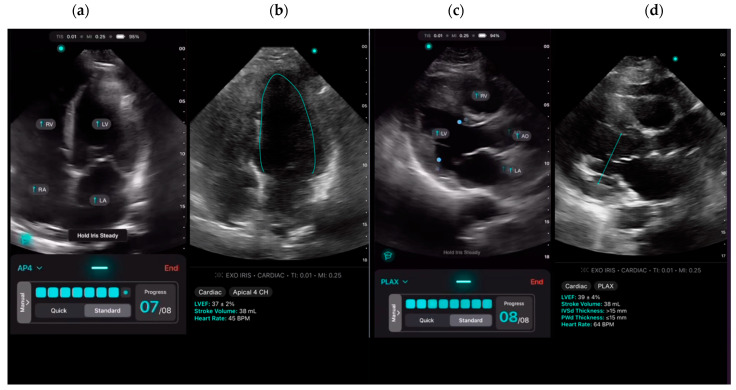
(**a**–**d**) Images were obtained using the AI-assisted tool on the Exo IRIS probe. (**a**) Apical 4 chamber (A4C) view, (**b**) AI-assisted LVEF of A4C, (**c**) Parasternal long axis view (PLAX), (**d**) AI-assisted LVEF of PLAX.

To assess the feasibility and inter-user reliability of the AI-assisted echo, we scanned a small cohort of patients (Cohort 1) multiple times. In these patients, the expert and all available novices separately and independently acquired their best attempts at PLAX and A4C views suitable for LVEF calculation using the AI-assisted tool on the Exo IRIS probe (Figure 1), positioning the probe based on real-time feedback from the algorithm. The time to achieve diagnostic-quality images was recorded for the experts and novices. If any novice learner was unable to obtain views of sufficient quality to generate AI LVEF estimates within 5 min, the expert would step in to verbally assist until images were captured.

To evaluate the accuracy of the AI-assisted echo, the expert then scanned a larger cohort of patients (Cohort 2), obtaining AI-assisted PLAX and A4C views in any patient who was also undergoing a contrast echocardiogram with quantitative LVEF calculation. This cohort did not involve novice learners performing scans. The gold-standard contrast echo performed by an expert sonographer in Cohort 2 patients was a complete conventional echo including (among other views) PLAX and A4C, performed without any AI guidance. All patients in Cohort 2 had this echo performed with contrast. Conventional echo scan was also performed in all Cohort 1 patients, but for logistical reasons, we were not able to administer contrast in all these patients. All gold standard scans were performed prior to AI-assisted POCUS scans and were therefore blinded to the AI-derived results.

### 2.7. Data Analysis

Data was systematically captured in the Exo Iris PACS system and a REDCap database. Collected metrics included scan times, image quality ratings, and LVEF classifications. Post-scan analysis involved AI-based LVEF calculations and expert evaluations of image quality and functionality.

### 2.8. Statistical Methods

ExoAI provides an estimated LVEF and a confidence interval with an upper and lower bound (Figure 1b,d).

We mainly focused our analysis on the mean estimate. Since the number of novice scanners varied between patients and each novice scanner was unique, a mean LVEF score from all novice scanners was calculated for each patient and used when relevant.

Non-parametric descriptive statistics were performed on continuous LVEF scores and scan times. Differences between expert and novice scanners and A4C and PLAX views in Cohort 1 were assessed using the Friedman test, while differences in LVEF between A4C and PLAX views acquired by the expert scanner in Cohort 2 were evaluated by Wilcoxon and McNemar’s tests. Inter-rater reliability of continuous LVEF measurements between expert and novice scanners was evaluated by intraclass correlation coefficient (ICC).

LVEF percentages were also converted to categorical data for normal/borderline (LVEF% > 40) and markedly reduced (LVEF % <= 40%). Inter-rater reliability of categorical assignment between expert and novice readers was assessed using Cohen’s kappa. The sensitivity and specificity of AI-extracted ultrasound LVEF vs. gold-standard classification, by contrast, echocardiography, were calculated.

## 3. Results

### 3.1. Feasibility

We averaged 3 novice learners per patient (range: 1 to 5) in Cohort 1. With 2 h of instruction and AI assistance, novice users were able to obtain images of sufficient quality for AI to measure left ventricle ejection fraction (LVEF) in nearly all patients. Out of 60 scans by novice readers on n = 10 patients, measurements could not be obtained by apical 4-chamber (A4C) view in 2 (6.7%) cases and by parasternal long axis view (PLAX) in 1 (3.3%) case.

The expert had similar rates of scan failure: Out of 80 patients scanned by the expert, measurements could be obtained by PLAX but not A4C in 3 (3.8%) cases and A4C but not PLAX in 3 (3.8%) cases.

**Figure 2:**
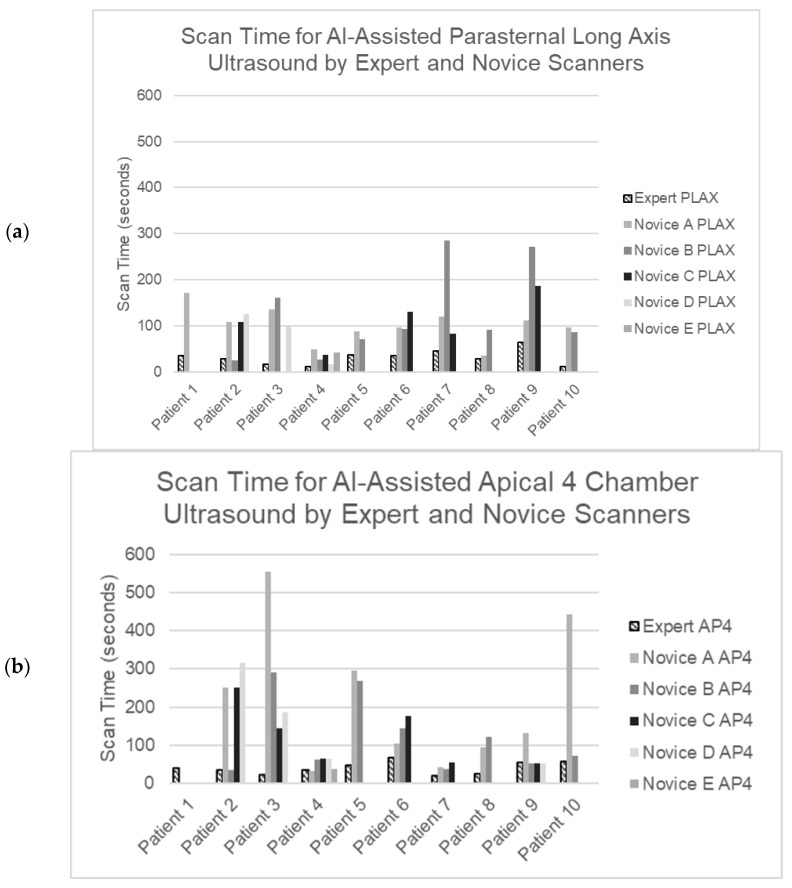
(**a**,**b**) Scan time for AI-assisted ultrasound by expert and novice scanners on n = 10 ICU patients for (**a**) parasternal long axis view and (**b**) apical 4 chamber view.

Scan times were significantly longer for novices than experts for both A4C and PLAX views (Figure 2a,b). Scan time for A4C: mean(IQR) 142(67–264) seconds for novices vs. 36(25–56) s for experts; PLAX: 92(76–140) s vs. 28(15–40) s; *p* < 0.00001.Scan times were less than 5 min for every PLAX scan and for all but 3 A4C scans. Although A4C scan times were often substantially longer than PLAX, for a given patient, the difference between the time to scan A4C and PLAX was not significant, either for experts or novices.

### 3.2. Reliability

Inter-rater reliability of continuous LVEF values between novice and expert scanners in Cohort 1 was high for both A4C and PLAX [ICC (95% CI)s: 0.88 (0.57–0.97) and 0.9 (0.67–0.97), respectively], and very high when considering the mean LVEF taken by averaging A4C and PLAX views [(ICC (95% CI):0.94 (0.77–0.99)] (Table 2).

**Figure 3:**
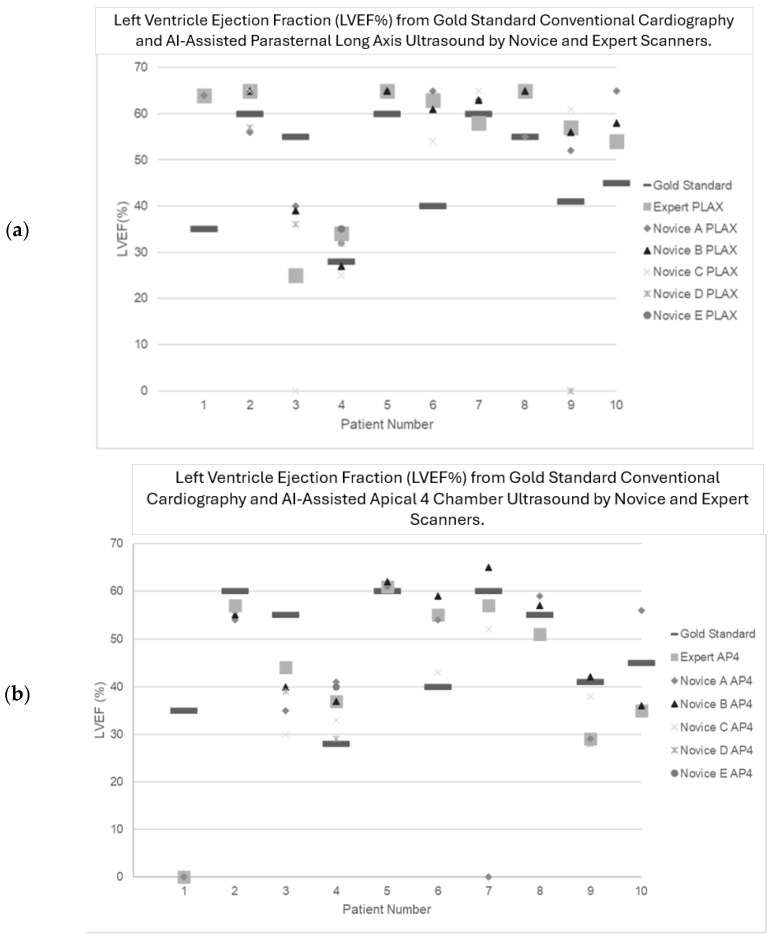
(**a**,**b**) Left Ventricle Ejection Fraction (LVEF%) from AI-assisted ultrasound by Expert and Novice Scanners for n = 10 ICU patients via (**a**) parasternal long axis view and (**b**) apical 4 chamber view.

When reviewing scan-by-scan data, the expert and novices generally had similar LVEF results, even when these diverged from the gold-standard conventionally measured LVEF (Figure 3a,b).

Considering LVEF classification by novice and expert raters at a threshold between normal/borderline (>40%) vs. markedly reduced (≤40%), there was perfect agreement between novices and experts using PLAX or the mean of A4C and PLAX [kappa (95% CI): 1.0 (1.0–1.0) for both], but only fair agreement when considering A4C values alone [kappa (95% CI) 0.5 (−0.10–1.0)].

### 3.3. Accuracy

We focused our evaluation of the accuracy of AI LVEF determination on Cohort 2, for whom a high-quality contrast-echo gold standard was consistently available. Reduced LVEF was present by gold-standard contrast echo in 27 (41.5%) of these cases.

LVEF values measured by AI on A4C and PLAX views vs. gold-standard in Cohort 2 are shown in Table 3a,b. We found LVEF values obtained via A4C were significantly lower than those obtained via PLAX [44% (35–55%) vs. 53% (34–58%), respectively, median (IQR), *p* = 0.042]. (Table 3a) This led to a slightly greater proportion of LVEF values being categorized by AI as reduced when measured from A4C than from PLAX (Table 3b). This was not statistically significant when considering AI mean values [24 (37%) cases for A4C vs. 19 (29%) for PLAX, *p* = 0.332)], but became significant for AI ‘lower bound’ values [33 (51%) vs. 20 (31%), *p* = 0.007]. Regardless of which view(s) were obtained, the AI generally detected fewer cases of reduced LVEF ≤ 40% (19–24 patients) than the gold-standard test did (27 patients).

**Chart 1:**
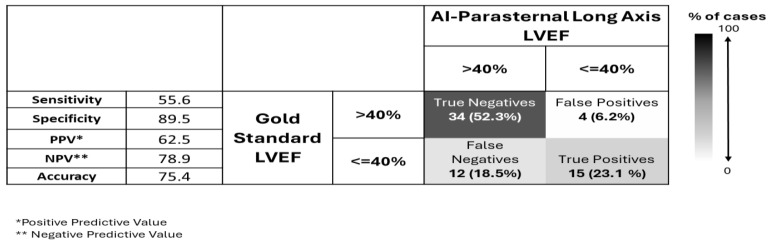
LVEF classification by AI-assisted parasternal long axis (PLAX) ultrasound compared to gold standard echocardiography in n = 65 expert-scanned cases.

**Chart 2:**
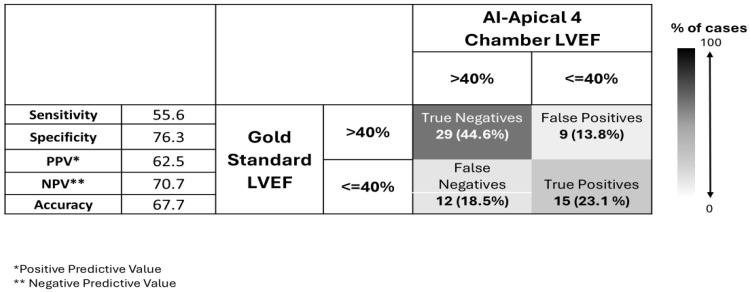
LVEF classification by AI-assisted apical 4 chamber (A4C) ultrasound compared to gold standard echocardiography in n = 65 expert-scanned cases.

**Chart 3:**
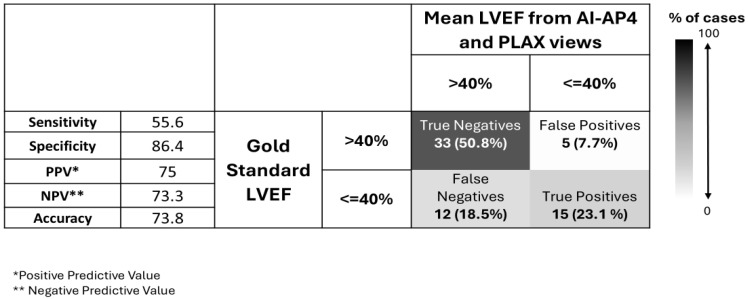
Mean LVEF classification by averaging LVEF measured from AI-assisted A4C and PLAX views compared to gold standard echocardiography in n = 65 expert-scanned cases.

**Chart 4:**
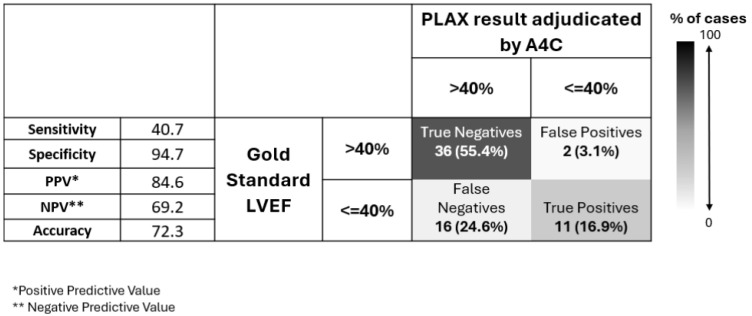
LVEF classification by AI-assisted ultrasound according to PLAX, adjudicated by A4C in cases where PLAX returned LVEF value ≤ 40% in n = 65 expert-scanned cases.

We computed confusion matrices for diagnostic strategies where the gold-standard LVEF is estimated clinically by performing only AI-PLAX, only AI-A4C, both, and a strategy where A4C is only added when LVEF on AI-PLAX view is reduced (Chart 1, Chart 2, Chart 3 and Chart 4).

From these confusion matrices, we see that AI-PLAX alone was highly specific for a reduced LVEF ≤ 40% (SP = 90%, PPV = 63%) but missed cases of reduced LVEF (SN = 56%). The AI-A4C view alone was equally sensitive (SN = 56%) and less specific (SP = 76%), conferring no advantage. Routinely combining AI-PLAX and AI-A4C views and averaging the LVEF obtained gave a profile fairly similar to just performing AI-PLAX alone. A strategy of performing AI-PLAX in all patients, performing AI-A4C and using the AI-A4C LVEF for classification only if the AI-PLAX found an abnormal LVEF was highly specific (SP = 95%, PPV = 85%) at the cost of sensitivity (SN = 41%). It was difficult to find an algorithm in which AI-PLAX and/or AI-A4C views could give high sensitivity for abnormal LVEF. If we used the AI “lower-bound” LVEF measurement rather than the mean, sensitivity did increase to 70%, 59%, and 70% (for A4C, PLAX, and mean of A4C + PLAX respectively), while mildly compromising specificity (to SP = 63%, 89% and 82% respectively).

### 3.4. Agreement and Bias

**Figure 4:**
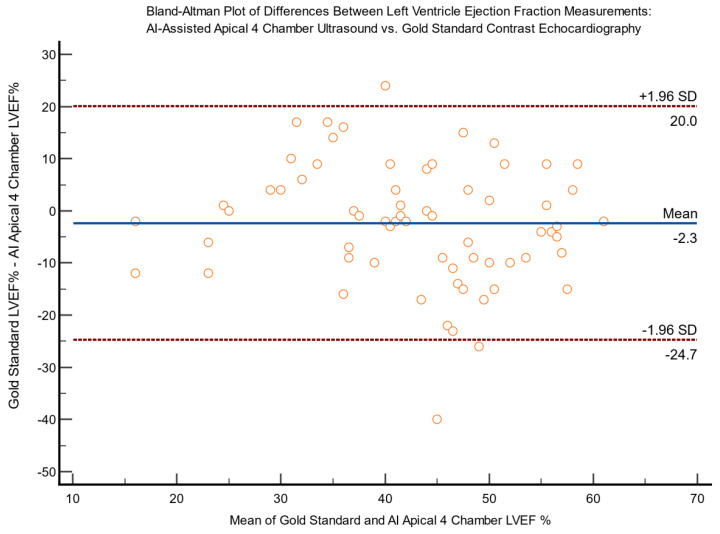
Bland-Altman Plot of differences between left ventricle ejection fraction (LVEF%) measurements via AI-assisted apical 4 chamber ultrasound (A4C) and gold standard contrast echocardiography (n = 65).

**Figure 5:**
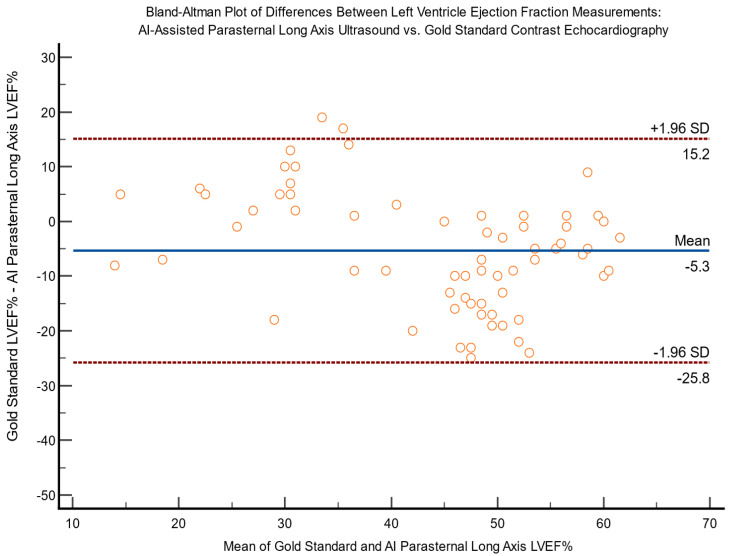
Bland-Altman Plot of differences between left ventricle ejection fraction measurements (LVEF%) via AI-assisted parasternal long axis ultrasound (PLAX) vs. gold standard contrast echocardiography (n = 65).

Bland-Altman plots for differences between expert AI-assisted ultrasound and gold standard echocardiography LVEF measurements are shown in Figure 4 and Figure 5. Some statistically significant bias can be seen in the mid-range of mean measurements (approximately 40–55%) from the gold standard and AI-PLAX (*p* = 0.0001), where AI-PLAX tends to produce higher measurements. This is consistent with the more limited sensitivity of AI-PLAX described in the previous section.

### 3.5. Accuracy in Cohort 1

Although Cohort 1 was designed to test reliability, we did also evaluate accuracy in this cohort. On the 10 patients who were scanned (a total of 80 times) by expert and multiple novices, mean LVEF on gold-standard echo was 50% (40–60%, mean(IQR)) and reduced LVEF (≤40%) values were present in 2 (22.2%). Median AI-generated LVEF values were not significantly different between novice and expert scanners nor between A4C and PLAX views (*p* > 0.05). This 10-patient cohort was too small for us to meaningfully evaluate diagnostic performance, but we did note that the SN and SP values in this cohort were identical for novice and expert scanners for both PLAX and A4C views.

### 3.6. Case-by-Case Measurement Comparison

Case-by-case LVEF measurements by gold standard contrast echocardiography as well as by expert and novice AI-assisted ultrasound are shown for both cohorts in (Supplementary Table S1).

## 4. Discussion

This prospective study investigated the feasibility, reliability and accuracy of determining the cardiac left ventricle ejection fraction (LVEF) from AI-enhanced ultrasound in an ICU setting. There are two related steps in this process: non-expert users must acquire diagnostic-quality images (with AI assistance) in these challenging patients, and AI must interpret the often-suboptimal images accurately. We had several key findings.

The feasibility of AI-enhanced echo was strong. We found that novice users with only 2 h of training could generate echo images of quality adequate for AI analysis in ~96% of ICU scans, with a 3–4% scan failure rate similar to that of experts. These results align with previous studies demonstrating that AI-enhanced ultrasound, utilized by both novice and expert users, consistently produces diagnostic quality images from the parasternal long axis (PLAX) and apical 4-chamber (A4C) views [13,15]. This result is particularly impressive considering the factors limiting echo in ICU patients: immobility (especially the inability to turn into decubitus position), potentially unstable clinical status, irregular and/or rapid heart rhythms, shadowing from abnormal lungs, large body habitus, uncooperative partially sedated or delirious patients, and artifacts from machinery such as mechanical ventilation.

While scan times were ~3× as long for novices as for experts, nearly all scans could be obtained in less than 5 min, even by novices. The PLAX view was easier and substantially faster for novices to obtain: their scan times averaged ~1.5 min for the PLAX view and ~3 min for A4C.

Inter-observer reliability was high. Novices and experts generated images that led to similar AI calculations of LVEF (ICC = 0.88–0.94 for the 2 views). This is expected since a key advantage of AI is that in many applications, it ‘levels the playing field’, enabling novices and experts to perform tasks nearly as well.

The accuracy of the AI LVEF calculations in these challenging ICU patients was more mixed. Concordant with frequent real-world practice [16], we used a threshold of 40% to differentiate between a substantially reduced LVEF (true-positive result) and a normal/minimally-reduced LVEF. When AI detected an LVEF ≤ 40%, it was generally correct, with a high specificity of 90–94%. The A4C view had more false-positive AI results than PLAX, potentially due to the increased difficulty of acquiring the A4C view and the effects of foreshortening in a suboptimal A4C view. Sensitivity was low at 56% when using the AI “mean LVEF” prediction, rising to 70% when using the AI “lower-bound LVEF” prediction. Overall, based on our observed test characteristics in ICU patients, this AI tool could be considered useful to confirm (i.e., “rule-in”) a suspected abnormal LVEF in the appropriate clinical context due to its high specificity and positive predictive value, but the AI tool would generally not confidently exclude (i.e., “rule-out”) decreased cardiac function.

When comparing A4C and PLAX views, while AI-derived measurements from the two views were generally similar, A4C LVEF estimates were significantly lower than those from PLAX in our cohort, leading to an underestimation of LVEF when relying on A4C alone. There is controversy in the literature regarding which view allows the most accurate AI-enhanced LVEF estimation, with some studies reporting that A4C outperforms PLAX [4,17] and other studies supporting our findings that PLAX was superior [14,18]. The differences may relate to patient cohorts and user experience. Since PLAX is more easily obtained by non-experts, these images may be of higher quality in more patients. However, because the PLAX view does not include the cardiac apex, patients with focal pathology affecting the apex (as is frequent in myocardial infarctions) may be best assessed by A4C views obtained by experts.

Given these discrepancies, many studies emphasize the value of integrating multiple echocardiographic views to improve the diagnostic accuracy of LVEF [4,14,17,18]. Consistent with this, we found that sensitivity and specificity for detecting reduced LVEF were highest when combining measurements from both A4C and PLAX. Performing the more easily obtained PLAX view in all patients and only adding A4C when PLAX was abnormal would have improved specificity slightly. Future larger studies could evaluate the validity of AI-enhanced single-view vs. multi-view approaches for LVEF assessment across different patient populations and clinical settings.

Our study had limitations. Although a strength of our study was the large number of novice scanners in an ICU setting (30 learners), we had only a small number of patients in Cohort 1 scanned by these novices (n = 10). This is because it was logistically difficult to have an ICU patient stable enough to be scanned by many learners when they were available. Again, we did not have a concurrent contrast-echo gold standard for all of these patients for logistical reasons. Another limitation is that the larger series in Cohort 2 (n = 65) assessing LVEF accuracy was scanned only by our expert, without learners; this is again due to logistical constraints in the hospital setting. However, since our results in Cohort 1 showed that the AI LVEF estimates were very similar whether the scan was performed by an expert or novice, the accuracy of the AI tool in Cohort 2 is likely to be broadly similar to that obtained by less-experienced users.

## 5. Conclusions

AI-enhanced echocardiography is feasible in ICU patients. After just 2 h of training, novices were able to obtain images of sufficient quality to produce an AI result in 96% of scans. The PLAX view took novices half as long to obtain as A4C, and both views could be obtained in <5 min. AI LVEF estimates were similar to those of whether the scan was performed by a novice or an expert. When AI detected a low LVEF, it was highly accurate, but AI had limited sensitivity for low LVEF, implying that this AI tool could “rule in”, but not as effectively “rule out”, a low LVEF. Caution is advised when the AI estimates LVEF to be normal in ICU patients.

This study highlights the potential of AI-enhanced ultrasound to improve cardiac assessment across diverse healthcare settings. In ICU, it could improve efficiency by streamlining evaluations, quickly confirming suspected poor cardiac function and reducing the burden on specialist sonographers. In areas lacking experts, such as small communities, AI-enhanced ultrasound could enable primary care providers to quickly conduct cardiac triage, facilitating timely referrals to specialist care for those most in need.

## Figures and Tables

**Table 1:** Characteristics of Cohorts 1 and 2, comprising consecutive patients receiving care in the ICU.

	Cohort 1	Cohort 2
	n = 10	n = 65
Expert Sonographer Data Available	Yes	Yes
Novice Sonographer Data Available	Yes	No
Gold Standard derived from Contrast Echo N (%)	2 (20%)	65 (100%)
Age Mean (SD)	57.6 (19.7)	64.3 (10.8)
Males N (%)	70%	71%
BMI Mean (SD)	27.9 (4.8)	27.2 (5.0)

**Table 2:** Intra-class correlation coefficients (ICC) for continuous AI-derived LVEF measurements obtained by expert vs. novice scanners from both A4C and PLAX views (n = 9 cases with all measurements available).

Measurement	ICC	95% CI
AI A4C	0.88	0.57–0.97
AI PLAX	0.9	0.67–0.97
Mean A4C and PLAX	0.94	0.77–0.99
AI A4C lower bound	0.85	0.50–0.96
AI PLAX lower bound	0.9	0.67–0.97
Mean A4C and PLAX lower bound	0.92	0.72–0.98

**Table 3:** (**a**) Descriptive statistics on cohort 2 data, n = 65 cases where gold standard and expert AI-generated LVEF data are available. The AI provides a mean and “lower bound” estimate of LVEF. (**b**) LVEF classification in cohort 2 data, n = 65 cases where gold standard and expert AI-generated A4C and PLAX ultrasound data are available.

(a)									
	Gold Standard		AI A4C		AI PLAX		AI A4C and PLAX		*p*-Value
	Median (IQR)	Range	Median (IQR)	Range	Median (IQR)	Range	Median (IQR)	Range	
LVEF% mean	42 (35–50)	10–63	44 (35–55)	17–65	53 (35–58)	12–65	49 (38–55)	20–63	0.042
LVEF % lower bound	-	-	40 (31–52)	15–62	50 (31–54)	7–63	60 (34–52)	16–60	0.062
(**b**)									
	**Gold Standard**		**AI A4C**		**AI PLAX**		**Mean AI A4C and PLAX**		*p*-Value *
	**N (%)**		**N (%)**		**N (%)**		**N (%)**		
LVEF ≤ 40%	27 (41.5%)		24 (36.9%)		19 (29.2%)		20 (30.8%)		0.3323
LVEF lower bound ≤ 40%	-		33 (50.8%)		20 (30.8%)		26 (40%)		0.0072

* for the difference between AI A4C and PLAX measurements.

## Data Availability

The datasets used and/or analyzed during the current study are available from the corresponding author upon reasonable request.

## Supplementary Materials

The following supporting information can be downloaded at: https://www.mdpi.com/article/10.3390/jcm14092899/s1, Table S1: Per-patient measurement of left ventricle ejection fraction (LVEF) % by gold standard contrast echocardiography, expert-acquired AI- assisted A4C and PLAX ultrasound, and novice-acquired AI-assisted A4C and PLAX ultra-sound.

## Abbreviations

The following abbreviations are used in this manuscript:

ICU	Intensive Care Unit
LVEF	Left Ventricular Ejection Fraction
PLAX	Parasternal Long Axis
A4C	Apical 4 Chamber
A2C	Apical 2 Chamber
AI	Artificial Intelligence
POCUS	Point-of-care Ultrasound
BMI	Body Mass Index
ICC	Intraclass Correlation Coefficient
SN	Sensitivity
SP	Specificity
PPV	Positive Predictive Value
NPV	Negative Predictive Value
