# Supplementary material for: AI-Augmented Point of Care Ultrasound in Intensive Care Unit Patients: Can Novices Perform a “Basic Echo” to Estimate Left Ventricular Ejection Fraction in This Acute-Care Setting?
Source: J Clin Med. 2025 Apr 23;14(9):2899. doi: 10.3390/jcm14092899 (PMC12072415; doi:10.3390/jcm14092899)
Supplement: Supplementary file 1 [file jcm-14-02899-s001.zip › jcm-3570779-supplementary.pdf]

Journal of
Clinical Medicine

Table S1. Per-patient measurement of left ventricle ejection fraction (LVEF) % by gold standard contrast echocardiography, expert-acquired AI- assisted A4C and PLAX ultrasound, and novice-acquired AI-assisted A4C and PLAX ultrasound. Duplicate of single gold standard measurement and single expert AI-assisted ultrasound measurement (per view) recorded per patient. Values are duplicated on multiple rows for comparison with multiple unique novice scanners

Case	Gold Standard	Expert PLAX	Expert A4C	Novice PLAX	Novice AP4
Patient 1 †	35	64	na	64	na
Patient 2	60	65	57	56	54
Patient 2	60	65	57	65	55
Patient 2	60	65	57	65	56
Patient 2	60	65	57	57	56
Patient 3	55	25	44	40	35
Patient 3	55	25	44	39	40
Patient 3	55	25	44	na	30
Patient 3	55	25	44	36	39
Patient 4	28	34	37	32	41
Patient 4	28	34	37	27	37
Patient 4	28	34	37	25	33
Patient 4	28	34	37	32	29
Patient 4	28	34	37	35	40
Patient 5	60	65	61	65	61
Patient 5	60	65	61	65	62
Patient 6	40	63	55	65	54
Patient 6	40	63	55	61	59
Patient 6	40	63	55	54	43
Patient 7	60	58	57	63	na
Patient 7	60	58	57	63	65
Patient 7	60	58	57	65	52
Patient 8	55	65	51	55	59
Patient 8	55	65	51	65	57
Patient 9	41	57	29	52	29
Patient 9	41	57	29	56	42
Patient 9	41	57	29	61	38
Patient 9	41	57	29	na	28
Patient 10	45	54	35	65	56
Patient 10	45	54	35	58	36
Patient 11	51	56	49	-	-
Patient 12	39	52	42	-	-
Patient 13	44	57	28	-	-
Patient 14	52	53	28	-	-
Patient 15	57	56	44	-	-
Patient 16	48	50	40	-	-
Patient 17	35	58	58	-	-
Patient 18	41	60	43	-	-
Patient 19	54	58	59	-	-
Patient 20	56	65	55	-	-
Patient 21	41	65	42	-	-
Patient 22	43	61	58	-	-
Patient 23	36	26	26	-	-
Patient 24	55	65	58	-	-
Patient 25	56	57	47	-	-
Patient 26	32	41	28	-	-
Patient 27	20	38	26	-	-
Patient 28	31	na	27	-	-
Patient 29	55	61	40	-	-
Patient 30	56	61	na	-	-
Patient 31	44	53	44	-	-
Patient 32	32	30	28	-	-

Case	Gold Standard	Expert PLAX	Expert A4C	Novice PLAX	Novice A4C
Patient 33	15	22	17	-	-
Patient 34	40	55	55	-	-
Patient 35	32	52	41	-	-
Patient 36	41	51	52	-	-
Patient 37	34	27	44	-	-
Patient 38	37	24	38	-	-
Patient 39	47	56	57	-	-
Patient 40	10	18	22	-	-
Patient 41	40	59	54	-	-
Patient 42	53	52	61	-	-
Patient 43	50	57	65	-	-
Patient 44	63	54	54	-	-
Patient 45	41	63	58	-	-
Patient 46	25	19	24	-	-
Patient 47	41	58	43	-	-
Patient 48	37	36	37	-	-
Patient 49	42	39	41	-	-
Patient 50	35	44	52	-	-
Patient 51	45	45	55	-	-
Patient 52	17	12	29	-	-
Patient 53	32	27	na	-	-
Patient 54	40	54	23	-	-
Patient 55	42	52	28	-	-
Patient 56	33	28	40	-	-
Patient 57	54	58	58	-	-
Patient 58	49	48	40	-	-
Patient 59	60	60	51	-	-
Patient 60	43	24	39	-	-
Patient 61	25	26	25	-	-
Patient 62	35	60	29	-	-
Patient 63	41	56	50	-	-
Patient 64	25	20	65	-	-
Patient 65	60	63	62	-	-
Patient 66	45	55	36	-	-
Patient 67	49	52	58	-	-
Patient 68	36	59	62	-	-
Patient 69	44	27	53	-	-
Patient 70	38	54	29	-	-
Patient 71	28	26	44	-	-
Patient 72	43	29	26	-	-
Patient 73	39	na	41	-	-
Patient 74	53	58	57	-	-
Patient 75	45	52	51	-	-
Patient 76	44	na	45	-	-
Patient 77	50	57	46	-	-
Patient 78	35	25	57	-	-
Patient 79	40	57	42	-	-
Patient 80	60	59	56	-	-

LVEF >40%
LVEF <=40%
no value obtained

†Patient 1 had fluctuating LVEF; gold standard measurement is questionable.
